# cGAS inhibition alleviates *Alu* RNA-induced immune responses and cytotoxicity in retinal pigmented epithelium

**DOI:** 10.1186/s13578-022-00854-y

**Published:** 2022-07-25

**Authors:** Jing Li, Feng Zhang, Wei Bian, Yanyun Chen, Jianying Liu, Zhenyu Liu, Ying Xiong, Xiuhua Wan

**Affiliations:** 1 Beijing Tongren Eye Center, Beijing Tongren Hospital of Capital Medical University, Beijing, 100730 China; 2 Department of Emergency Medicine, Affiliated Hospital of Sergeant School Affiliated to Army Medical University, Shijiazhuang, 050000 China

**Keywords:** RPE, AMD, GA, cGAS, *Alu* RNA, EGCG, RSVL

## Abstract

**Background:**

The degeneration of retinal pigmented epithelium (RPE) cells results in severe diseases, such as age-related macular degeneration (AMD) that causes blindness in millions of individuals.

**Results:**

We report that targeting GMP-AMP (cGAMP) synthase (cGAS) alleviates *Alu* RNA-induced immune responses and cytotoxicity in RPE. We find that the deletion of cGAS in RPE inhibits the *Alu* RNA-stimulated interferon production. cGAS deficiency also protects RPE from cell death triggered by *Alu* RNA. Importantly, two natural chemicals, epigallocatechin gallate (EGCG) and resveratrol (RSVL), are effective in suppressing the immunogenic and cytotoxic effect of *Alu* RNA in RPE.

**Conclusions:**

Our findings further demonstrate the crucial role of cGAS in the *Alu* RNA-induced RPE damage and present EGCG and RSVL as potential therapies for AMD and other RPE degeneration-related conditions.

## Introduction

Age-related macular degeneration (AMD) is a prevalent disease that causes blindness in aged individuals [1–3]. Millions of people worldwide are suffering from vision loss as a result of AMD [1–3]. There are two main forms of AMD, neovascular AMD and geographic atrophy (GA) [1, 2, 4, 5]. The neovascular AMD has been effectively treated through anti-angiogenesis strategies, such as targeting the vascular endothelial growth factor A (VEGFA) [1, 2, 5–7]. In contrast, GA, the advanced form of AMD, is still untreatable [2, 4, 5, 8–12]. RPE cells form a monolayer of tissue that play critical roles in supporting the homeostasis of retina [2–4]. Studies indicated that the RPE degeneration is not only a key characteristic manifestation of GA, but also a major pathological factor that triggers GA [1, 4, 9, 10]. Therefore, understanding the mechanisms underlying the degeneration of RPE is critical for the development of therapies for GA.

*Alu* RNA is a type of noncoding RNAs transcribed from *Alu* elements, which are the most abundant repetitive elements in the genome of humans [13–15]. It is believed that there are about 1 million copies of *Alu* elements in each genome [13, 15]. A growing number of evidence showed that these repetitive elements shape the genome both structurally and functionally [13, 15]. Interestingly, recent studies illustrated the pathogenic role of *Alu* RNAs in GA and found that the accumulation of *Alu* RNAs in RPE induced cell death through the activation of inflammasome [14] and the cytosolic DNA sensor, cGAS [9].

As a primary intracellular DNA sensor, cGAS detects the cytosolic DNA to elicit the downstream immune responses, such as the production of type I interferons (IFNs) [16–18]. The emergence of DNA in the cytoplasm can be a result of either cellular damage or microbial infections [19]. *Alu* RNAs were found to activate cGAS by inducing the release of mitochondrial DNA (mtDNA), which is a ligand for cGAS [9, 20]. cGAS activation then drives the inflammasome activation and the RPE degeneration in AMD [9]. Thus, the activation of cGAS is an important upstream event during the *Alu* RNAs-induced RPE degeneration. It is therefore suggested that cGAS inhibition may be a potential mean to preserve RPE health and to treat GA. Several cGAS inhibitors were identified recently. For example, two natural chemicals, epigallocatechin gallate (EGCG) [16] and resveratrol (RSVL) [21], were showed to suppress cGAS activation efficiently. In the current study, we explored whether these cGAS-inhibiting reagents could be used to ameliorate the *Alu* RNAs-induced immune responses and cell death in RPE.

## Results

### dsRNA induced the interferon production in RPE cells

**Fig.1:**
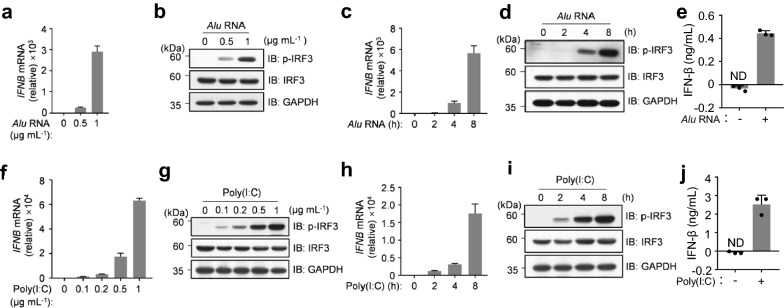
dsRNA induced the interferon production in ARPE-19 cells. **a**, **b** ARPE-19 cells were transfected with *Alu* RNA (0.5 μg mL^−1^ or 1 μg mL^−1^) for 8 h. *IFNB* mRNA levels was analyzed by qPCR (**a**) and immunoblot analysis of indicated proteins (**b**). **c**, **d** ARPE-19 cells were transfected with *Alu* RNA (1 μg mL^−1^) for indicated hours and *IFNB* mRNA levels was analyzed by qPCR (**c**), immunoblot analysis of indicated proteins (**d**). **e** ARPE-19 cells were transfected with *Alu* RNA (1 μg mL^−1^) for 24 h and the secreted IFN-β was measured by ELISA. **f**, **g** ARPE-19 cells were transfected with poly(I:C) as indicated concentrations for 4 h and *IFNB* mRNA levels was analyzed by qPCR (**f**), and the expression of indicated proteins were analyzed with immunoblotting (**g**). **h**, **i** ARPE-19 cells were transfected with poly(I:C) (0.2 μg mL^−1^) as indicated and *IFNB* mRNA levels was analyzed by qPCR (**h**). The expression of indicated proteins were analyzed with immunoblotting (**i**). **j** ARPE-19 cells were transfected with poly(I:C) (1 μg mL^−1^) for 24 h and the secreted IFN-β was measured by ELISA. Data are mean ± s.e.m. of three independent experiments (**e** and **j**) and mean ± s.e.m. of three technical repeats (**a**, **c**, **f** and **h**). ND, not detected (**e** and **j**). GAPDH (**b**, **d**, **g** and **i**), loading controls. Data are representative of at least two independent experiments

*Alu* RNA accumulation is implicated in GA [14]. To mimic this condition, we synthesized *Alu* RNA transcripts and transfected ARPE-19, an RPE cell line, with *Alu* RNAs. As expected, the introducing of *Alu* RNAs into the cytoplasm of ARPE-19 cells triggered the robust expression of IFN (Fig. 1a). The intracellular nucleic acid-stimulated expression of IFN is dependent on the transcriptional factor, interferon regulatory factor 3 (IRF3) and the phosphorylation level of IRF3 can be used to reflect its activation [22–24]. We then detected the phosphorylation of IRF3 using immunoblotting with the specific antibodies against the phosphorylated IRF3. We showed that the transfection of *Alu* RNAs strongly stimulated the activation of IRF3 (Fig. 1b). We also observed the *Alu* RNA-induced IFN expression at different time points post transfection and found that *Alu* RNAs triggered the expression of IFN in a time-dependent manner (Fig. 1c, d). We next confirmed these findings by measuring the production of IFN using enzyme-linked immunosorbent assay (ELISA) (Fig. 1e). Further, using the synthetic analog of double-stranded RNA (dsRNA), polyinosinic-polycytidylic acid [poly(I:C)], we obtained the consistent results (Fig. 1f–j). Together, these data suggested that *Alu* RNAs and other dsRNAs induce the interferon production in RPE cells.

### cGAS is required for *Alu* RNA-induced IFN expression

**Fig.2:**
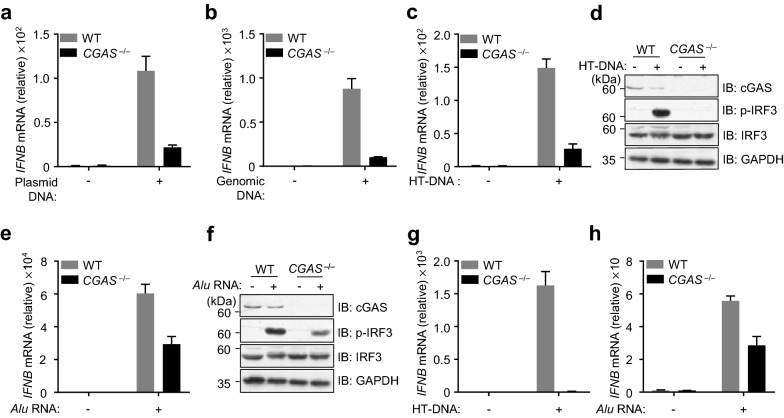
cGAS is critical for *Alu* RNA-induced interferon expression. **a**–**c** WT and *CGAS*^*−/−*^ ARPE-19 cells were transfected with plasmid DNA (4 μg mL^−1^) for 6 h (**a**), genomic DNA (4 μg mL^−1^) for 6 h (**b**) or HT-DNA (2 μg mL^−1^)for 6 h (**c**) and *IFNB* mRNA levels were analyzed by qPCR. **d** Immunoblot analysis of indicated proteins in WT and *CGAS*^*−/−*^ ARPE-19 cells that transfected with HT-DNA. **e** WT and *CGAS*^*−/−*^ ARPE-19 cells were transfected with *Alu* RNA (1 μg mL^−1^) for 8 h and *IFNB* mRNA levels was analyzed by qPCR. **f** Immunoblot analysis of indicated proteins in WT and *CGAS*^*−/−*^ ARPE-19 cells that transfected with *Alu* RNA. **g**, **h** WT and *CGAS*^*−/−*^ U937 cells were transfected with HT-DNA (2 μg mL^−1^) (**g**) or *Alu* RNA (1 μg mL^−1^) (**h**) for 3 h and *IFNB* mRNA levels was analyzed by qPCR. Data are mean ± s.e.m. of three technical repeats (**a**, **b**, **c**, **e**, **g** and **h**). GAPDH (**d** and **f**), loading controls. Data are representative of at least two independent experiments

As previous publication indicated that the *Alu* RNA stimulates cGAS activation through inducing the release of mtDNA [9], we next tested this in cells that we studied. We first generated *cGAS* null RPE cells using CRISPR/Cas9. As expected, cGAS deletion abolished different types of DNA-induced IFN expression (Fig. 2a–c). By detecting the HT-DNA-induced phosphorylation of IRF3, we obtained consistent results (Fig. 2d). We then transfected both wild-type (WT) and *cGAS*^–/–^ ARPE-19 cells with *Alu* RNAs and confirmed that cGAS is required for *Alu* RNA-induced IFN expression (Fig. 2e, f). We also used the WT and *CGAS*^–/–^ U937 cells, the monocytic cell line that is widely used for cGAS study, to examine the role of cGAS in response to *Alu* RNA challenge. In U937 cells, cGAS deficiency disrupted the DNA-induced IFN expression (Fig. 2g). The cGAS-mediated response to *Alu* RNA seemed to be a universal mechanism, as the *Alu* RNA-stimulated IFN expression was also attenuated in cGAS null U937 cells (Fig. 2h). Thus, cGAS is a key mediator in the signaling pathway downstream of *Alu* RNA in RPE.

### cGAS is critical for *Alu* RNA-induced RPE death

**Fig.3:**
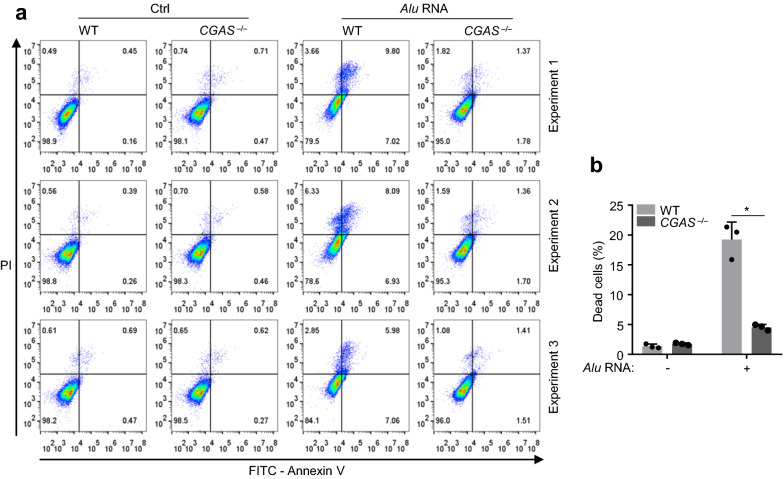
cGAS is critical for *Alu* RNA-induced cell death. **a** Annexin V/PI analysis by flow cytometry of WT and *CGAS*^*−/−*^ ARPE-19 cells transfected with *Alu* RNA (4 μg mL^−1^) for 48 h or non-transfected (Ctrl). **b** The percentage of dead cells in (**a**) was quantified. Data are mean ± s.e.m. of three independent experiments, *P < 0.05, two-tailed *t*-test (**b**)

To assess the *Alu* RNA-induced cell death in ARPE-19 cells, we transfected WT and *cGAS*^–/–^ cells with *Alu* RNAs and harvested the cells 48 h post transfection. The cells were then stained with Annexin V and propidium iodide (PI), which respectively indicate the early apoptotic cell death and the late apoptotic or other forms of cell death [25]. Using flow cytometry, we analyzed the percentage of dead cells in the transfected ARPE-19. We showed that while cGAS deletion did not lead to detectable cell death, it significantly reduced the *Alu* RNA-induced death of ARPE-19 (Fig. 3a, b). Thus, cGAS deficiency may prevent RPE from *Alu* RNA-induced cell death. Our data further suggested that inhibition of cGAS could be used to rescue the *Alu* RNA-associated RPE degeneration.

### EGCG/RSVL inhibits cGAS activation

**Fig.4:**
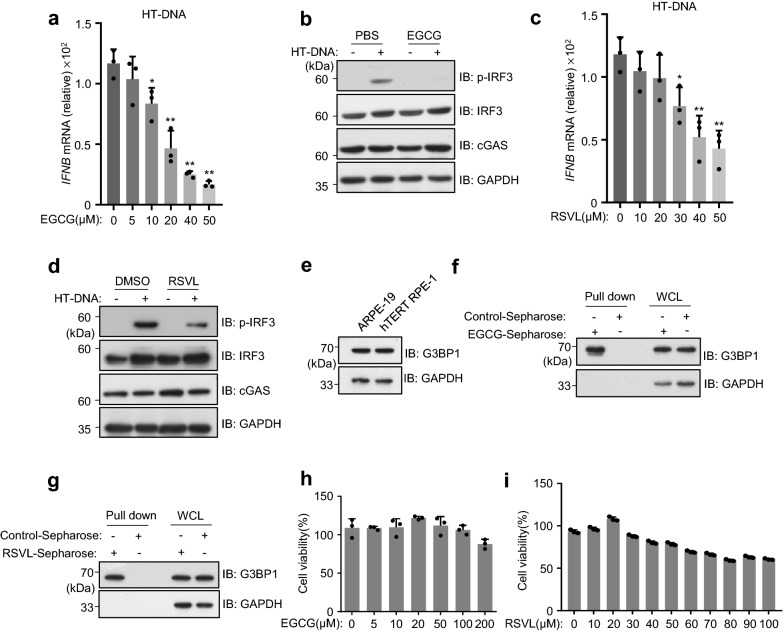
EGCG/RSVL inhibits cGAS activation.** a**–**d** ARPE-19 cells were transfected with HT-DNA (2 μg mL^−1^) for 6 h, following a 3-h pretreatment with EGCG or RSVL as indicated, qPCR analysis of *IFNB* mRNA levels (**a** and **c**). Immunoblot analysis of expression levels of proteins, EGCG (20 μM) or RSVL (40 μM) were respectively used (**b** and **d**). **e** Immunoblot analysis of indicated proteins of ARPE-19 and hTERT RPE-1. **f**, **g** Pull-down assays with EGCG/RSVL-conjugated Sepharose beads in ARPE-19 cells. EGCG/RSVL-binding proteins were analyzed by immunoblotting with indicated antibodies (**f** and **g**). **h**, **i** Cell viability of ARPE-19 cells treated by EGCG (**h**) or RSVL (**i**) as indicated. Data are mean ± s.e.m. of three independent experiments (**a**, **c**, **h** and **i**), *P < 0.05, **P < 0.01, two-tailed *t*-test (**a** and **c**). GAPDH (**b, d, e, f** and **g**), loading controls. Data are representative of at least two independent experiments

EGCG and RSVL were respectively showed to inhibit the activation of cGAS [16, 21]. We therefore examined their effects in ARPE-19 cells. To do so, ARPE-19 cells were pretreated with either EGCG or RSVL, followed by the transfection of DNA, which specifically activates cGAS. Our results showed that the pretreatment of EGCG significantly suppressed the DNA-induced IFN expression in RPE cells (Fig. 4a). We also showed that EGCG effectively blocked the DNA-triggered phosphorylation of IRF3 (Fig. 4b). Similarly, we further showed that the pretreatment of RSVL also led to the inhibition of DNA-induced cGAS activation (Fig. 4c, d). Because EGCG and RSVL were reported to inhibit cGAS activation through GTPase-activating protein SH3 domain–binding protein 1(G3BP1) [16, 21], we confirmed the expression of G3BP1 in both ARPE-19 and hTERT RPE-1 cell lines (Fig. 4e). Further, using EGCG- and RSVL-conjugated Sepharose beads, we performed pull-down assays and showed that both EGCG and RSVL can selectively bind to G3BP1 protein (Fig. 4f, g).

We next examined the cytotoxicity of both EGCG and RSVL on ARPE-19 cells. To do so, we cultured ARPE-19 cells in the presence of EGCG or RSVL for 48 h and analyzed the cell death using CellTiter assays. Our data showed that EGCG did not induce obvious cell death at 200 μM (Fig. 4h), while it significantly suppressed cGAS at 20 μM (Fig. 4a). RSVL exhibited marginal toxic effect on ARPE-19 cells (Fig. 4i). Thus, both EGCG and RSVL can be tolerated by ARPE-19 cells, at the concentrations we used to inhibit cGAS activation.

### EGCG/RSVL suppresses *Alu* RNA-induced IFN expression

**Fig.5:**
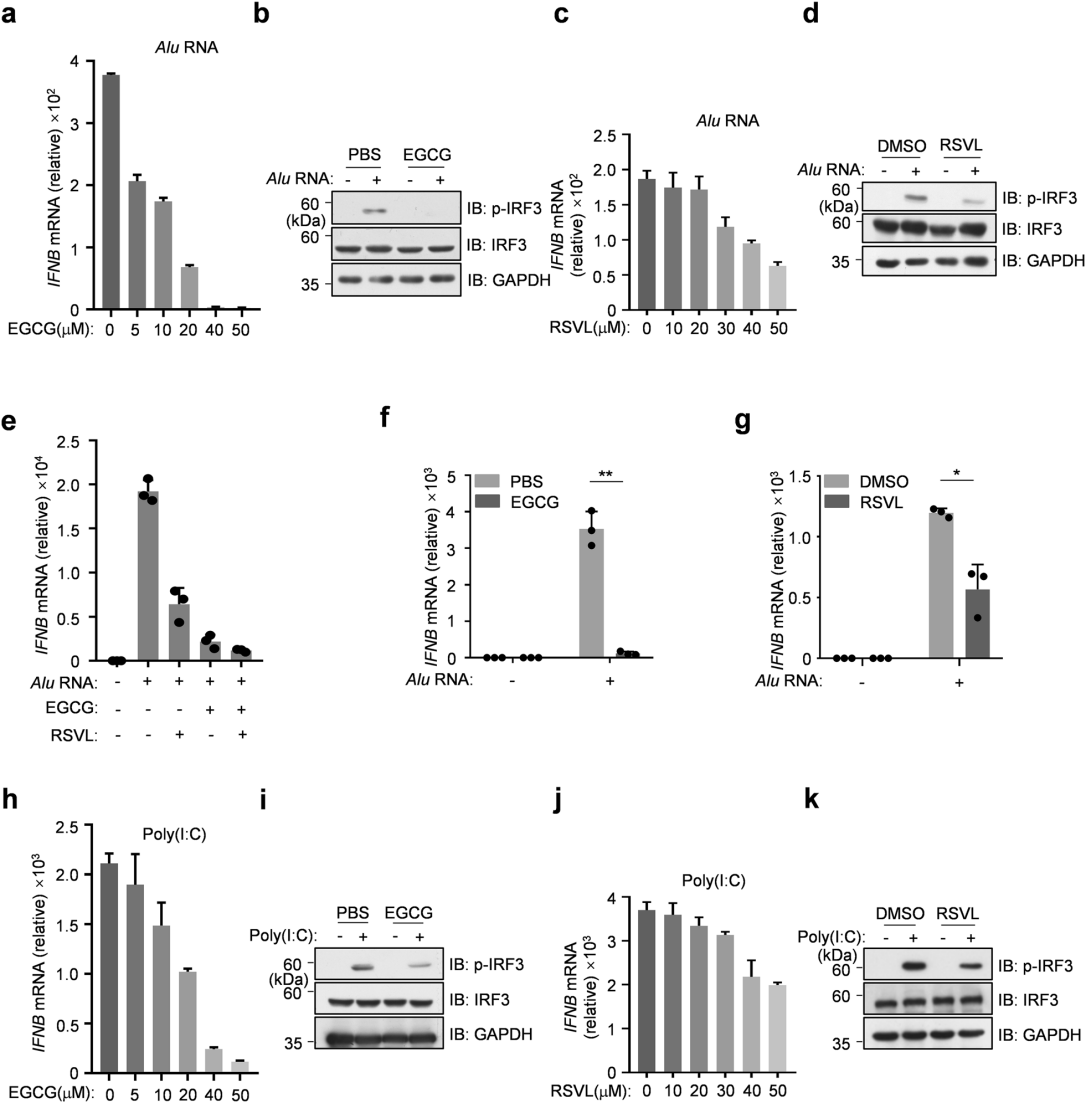
EGCG/RSVL suppresses *Alu* RNA-induced interferon expression.** a**–**e** ARPE-19 cells were transfected with *Alu* RNA (1 μg mL^−1^) for 8 h, following a 3-h pretreatment with EGCG or RSVL as indicated. qPCR analysis of *IFNB* mRNA levels (**a, c** and **e**). Immunoblot analysis of indicated proteins, EGCG (20 μM) or RSVL (40 μM) were respectively used (**b** and **d**). **f**, **g** hTERT RPE-1 cells were transfected with *Alu* RNA (1 μg mL^−1^) for 8 h, following a 3-h pretreatment with EGCG or RSVL as indicated. qPCR analysis of *IFNB* mRNA levels. **h–k** ARPE-19 cells were transfected with poly(I:C) (0.2 μg mL^−1^) for 4 h, following a 3-h pretreatment with EGCG or RSVL as indicated. qPCR analysis of *IFNB* mRNA levels (**h** and **j**). Immunoblot analysis of indicated proteins, EGCG (20 μM) or RSVL (40 μM) were respectively used (**i** and **k**). Data are mean ± s.e.m. of three technical repeats (**a**, **c**, **h** and **j**) and mean ± s.e.m. of three independent experiments (**e**, **f** and **g**), *P < 0.05, **P < 0.01, two-tailed *t* test (**f** and **g**). GAPDH (**b**, **d**, **i** and **k**), loading controls. Data are representative of at least two independent experiments

We then tested the effect of EGCG on *Alu* RNA-transfected RPE cells. When the cells were pretreated with increasing amount of EGCG followed by the *Alu* RNA transfection, we found that EGCG effectively dampened *Alu* RNA-induced IFN expression at 5 μM. Strikingly, 40 μM of EGCG almost blocked IFN expression triggered by *Alu* RNA (Fig. 5a). Consistently, EGCG treatment inhibited the *Alu* RNA-induced phosphorylation of IRF3 (Fig. 5b). Although the inhibitory effect of RSVL was not as potent as EGCG, our data showed that RSVL can markedly reduce the *Alu* RNA-induced IFN expression and the activation of IRF3 (Fig. 5c, d). We also treated the cells with EGCG and RSVL together to explore whether there was a synergistic effect of these two chemicals. As shown in Fig. 5e, EGCG + RSVL did not obviously inhibit the *Alu* RNA-induced IFN expression further, probably because the effect of EGCG alone was efficient enough. Moreover, with hTERT RPE-1 cells, we confirmed the effect of EGCG and RSVL (Fig. 5f, g). Using poly(I:C), we obtained the similar data indicating that both EGCG and RSVL were effective in attenuating dsRNA-induced IFN expression (Fig. 5h–k). Taken together, both EGCG and RSVL can be used to inhibit the dsRNA-triggered IFN expression.

### EGCG/RSVL restrained *Alu* RNA-induced cell death of RPE

**Fig.6:**
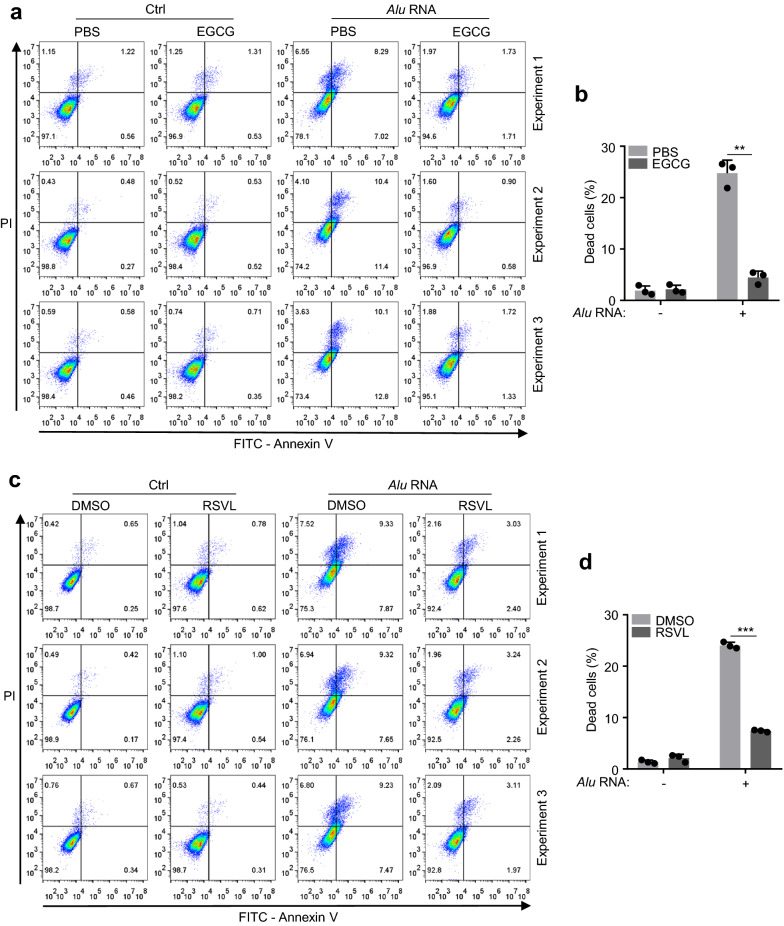
EGCG/RSVL suppresses *Alu* RNA-induced cell death. **a** Annexin V/PI analysis by flow cytometry of ARPE-19 cells transfected with *Alu* RNA (4 μg mL^−1^) for 48 h or non-transfected (Ctrl), following a 3-h pretreatment of EGCG (20 μM). **b** The percentage of dead cells in (**a**) was quantified. **c** Annexin V/PI analysis by flow cytometry of ARPE-19 cells transfected with *Alu* RNA (4 μg mL^−1^) for 48 h, following a 3-h pretreatment of RSVL (40 μM). **d** The percentage of dead cells in (**c**) was quantified. Data are mean ± s.e.m. of three independent experiments, **P < 0.01, ***P < 0.001, two-tailed *t*-test (**b** and **d**)

As *Alu* RNA significantly induced cell death in ARPE-19 cells (Fig. 3a, b) and cGAS deficiency prevented such cell death (Fig. 3a, b). We reasoned that EGCG and RSVL may have the effect in restraining *Alu* RNA-induced cell death in RPE cells. We then verified our hypothesis by treating ARPE-19 cells with EGCG prior to the *Alu* RNA-transfection. Our data showed that EGCG significantly recured the cell death triggered by *Alu* RNA transfection (Fig. 6a, b). RSVL also showed a similar effected in preventing cell death of RPE in the condition of *Alu* RNA challenging (Fig. 6c, d).

Taken together, our data suggested that inhibition of cGAS by EGCG or RSVL could be a potential treatment for *Alu* RNA-induced RPE degeneration. Our study thereby presenting these natural chemicals as possible therapies for GA.

## Discussion

AMD, especially the advanced form, GA, is a prevalent, severe, and currently untreatable disease that causes vision-loss in millions of individuals [3, 8, 14, 26]. The degeneration of RPE cells has been known as a major player in the pathogenesis of GA [3, 8, 14, 26]. However, the lack of detailed molecular mechanisms of the RPE degeneration has hampered the development of effective therapies for GA [1]. Recently, a series of exciting works highlighted the critical role of cGAS in the *Alu* RNA-induced RPE death [8, 9, 14]. In the current study, we showed that inhibition of cGAS with natural chemicals protected RPE from *Alu* RNA-triggered cell death. We first showed that the deletion of cGAS in RPE dampened the *Alu* RNA-stimulated interferon production. cGAS-deficient RPE cells were resistant to *Alu* RNA-induced cell death. Importantly, we found that two natural chemicals, EGCG and RSVL, were effective in suppressing the immunogenic and cytotoxic effect of *Alu* RNA on RPE. Thus, our findings further demonstrated the crucial role of cGAS in the *Alu* RNA-induced RPE damage and present EGCG and RSVL as potential treatments for RPE degeneration-related conditions, such as AMD.

cGAS is a cytoplasmic DNA sensor that responsible for the detection of invading pathogens by sensing the emerging of DNAs in the cytosol [19]. The aberrant activation of cGAS by self-DNA can be a major cause for a type of human diseases [16, 18]. For example, the insufficient clearance of self-DNAs derived from the transcription of endogenous retroviruses or retrotransposons led to the accumulation of self-DNAs in the cytoplasm, which chronically stimulate the activation of cGAS [18, 27]. In RPE cells, elevated transcription of *Alu* element results in the release of mtDNAs, which activate cGAS and its downstream production of interferons, and the activation of cGAS is required for the further activation inflammasome [8, 9, 14]. These events finally caused the degeneration of RPE cells. Our data suggested that the *Alu* RNA-mtDNA release-cGAS activation could be a universal mechanism in different cells. Thus, cGAS is a key target for the treatment of many intracellular nucleic acid-related diseases.

NLRP3 Inflammasome is a key molecular machinery assembled in response to a variety of stimuli [28–31]. Besides the infection-related danger signal molecules, such as nigericin [30, 32–34] and double stranded RNAs [35], the endogenous damaged-associated molecules were also found to trigger the activation of NLRP3 inflammasome [35, 36]. The activation of inflammasomes, including NLRP3 inflammasome, is essential for the secretion of pro-inflammatory cytokines, interleukin (IL)-1β and IL-18, and the inducing of inflammation-prone cell death called pyroptosis [36–40]. The aberrant activation of NLRP3 inflammasome was found in many human diseases, including Alzheimer’s diseases, type 2 diabetes, and gout [14, 39, 41–47]. Notably, in addition to inhibiting the activation of cGAS, EGCG was also reported to suppresses the activation of NLRP3 inflammasome [16, 48–51]. Moreover, a recent report showed that EGCG can also attenuate the mtDNA synthesis and thereby block NLRP3 inflammasome activation [48].

Besides *Alu* RNA-mtDNA releasing pathway, *Alu* RNA may also trigger the intracellular RNA sensor-mediated immune responses. EGCG and RSVL were recently reported to block intracellular RNA-sensing signaling [21]. The inhibitory effect of these two chemicals were mainly attributed to the inhibition of a key factor, G3BP1 [16, 21, 52]. Interestingly, G3BP1 was also a core organizer for the assembly of stress granules (SG), which is an important molecular condensation assembled in response to stress signals, such as the emergence of irregular RNA molecules in the cytoplasm [16, 53–55]. Although we did not detect the formation of SGs upon *Alu* RNA challenges in our study, it is very likely that *Alu* RNA will trigger the assembly of SG. Thus, through targeting G3BP1, EGCG and RSVL could preserve RPE health by executing the inhibitory effects at multiple layers of the dysregulated immune responses during RPE degeneration. As natural chemicals, both EGCG and RSVL are abundant in nature and are easy to acquire from plants [16, 21, 56]. Our study therefore suggests these chemicals as tangible lead compounds to prevent the development of GA.

## Conclusions

Our findings further demonstrate the crucial role of cGAS in the *Alu* RNA-induced RPE damage and present EGCG and RSVL as potential therapies for AMD and other RPE degeneration-related conditions.

## Materials and methods

### Reagents

Anti-p-IRF3 (ab76493) and anti-IRF3 (ab68481) were from Abcam; EGCG (E4143), RSVL (R5010) and HT-DNA (D6898) were from Sigma-Aldrich; Poly(I:C) (tlrl-pic) was from InvivoGen; Anti-G3BP1 (13057-2-AP) was from Proteintech Group; Plasmid DNA, used as the DNA stimulator, was an empty vector plasmid (pCDX-Tet-On) and amplified with PureYield Plasmid Midiprep System (A2492, Progema); Genomic DNAs were purified using StarSpin Animal DNA Kit (D111-01, GenStar); Anti-human cGAS and anti-human GAPDH antibodies were gifts from Dr. Tao Li at National Center of Biomedical Analysis, Beijing, China.

### Cell culture and transfection

ARPE-19 cells were cultured in Advanced DMEM/F12 medium containing 10% Fetal Bovine Serum, 2 mM glutamine, 100 mg mL^−1^ penicillin, 100 mg mL^−1^ streptomycin. Cells were grown in a 5% CO_2_ incubator (Thermo Fisher Scientific) at 37 °C. All cell lines were tested to be mycoplasma free by PCR.

Transfection of poly(I:C), HT-DNA and *Alu* RNA were performed with Lipofectamine 2000 (Invitrogen).

### Cell viability assay

ARPE-19 cells were seeded into 96-well plates and incubated with EGCG or RSVL at indicated concentrations for 48 h. CellTiter 96^®^ AQueous One Solution Cell Proliferation Assay (G3580, Promega) was performed to analyze the cell viability according to the manufacturer’s instruction.

### Annexin V and PI staining

WT and *CGAS*^*−/−*^ ARPE-19 cells were transfected with *Alu* RNA (4 μg mL^−1^) for 48 h, with or without a 3-h pretreatment of EGCG or RSVL. The Annexin V- and PI-positive cells were then measured by flow cytometer (BD Accuri^tm^ C6 Plus analyzer) using the Annexin V-FITC apoptosis detection kit (P04D03, Gene-Protein Link).

### RNA extraction and quantitative PCR (qPCR)

Total RNAs were isolated from cells with TRIZOL reagent (93,289, Sigma-Aldrich) and reverse transcribed with PrimeScript RT Reagent Kit (TaKaRa, RR037A). qPCR was performed with Powerup SYBR Green Master Mix (A25742, Thermo Fisher Scientific) on an ABI StepOnePlus system according to the manufacturer’s instructions. qPCR data was analyzed by StepOnePlus software. The sequences for qPCR primers are listed below. mRNA level of human *GAPDH* was used for normalization.

Human *IFNB*, sense: AGGACAGGATGAACTTTGAC,

anti-sense: TGATAGACATTAGCCAGGAG.

Human *GAPDH*, sense: GAGTCAACGGATTTGGTCGT,

anti-sense: TTGATTTTGGAGGGATCTCG.

### CRISPR/Cas9-mediated gene knockout in cells

For targeting *CGAS* with CRISPR/Cas9 in ARPE-19 cells, we used a LentiCRISPR v2 construct (Addgene, #98290). The single guide RNA (sgRNA) sequences of *CGAS* (sg-h*CGAS*: 5ʹ-CACCGAAGTGCGACTCCGCGTTCAG-3′) was designed using website of Dr. F. Zhang’s lab (http://crispr.mit.edu/). The lentiCRISPR-sgRNA was co-transfected with psPAX2 (Addgene, #12260) and pVSVg (Addgene, #8454) into HEK293T cells for 48 h to generate lentivirus. ARPE-19 cells were infected with lentivirus for 48 h, followed by culturing with puromycin (2 μg mL^−1^) for 7 days. Protein expression was analyzed by Western blotting.

### Immunoblotting

Cells were lysed with lysis buffer (20 mM Tris–HCl pH 7.5, 0.5% Nonidet P-40, 250 mM NaCl, 3 mM EDTA, 3 mM EGTA, 2 mM dithiothreitol) with protease inhibitor cocktail (Roche, 04,693,132,001). Cell lysates were separated by SDS-PAGE and proteins were transferred onto PVDF membranes. The transferred PVDF membranes were blocked by 5% milk for 1 h at room temperature and subjected to primary antibody incubation at 4℃ for overnight. Protein bands were visualized with enhanced chemiluminescence (ThermoFisher Scientific).

### *Alu* RNA transcription

*Alu* RNA were transcribed using MEGAshortscript™ Kit (AM1354) in vitro according to the manufacturer’s instructions.

### Pull-down assay

EGCG/RSVL was conjugated with cyanogen bromide (CNBr)-activated Sepharose 4B (GE Healthcare). Cells were lysed in lysis buffer (Tris–HCl 20 mM, pH 7.5; NaCl 10 mM; 0.5% Nonidet P-40; EDTA 3 mM, EGTA 3 mM) with protease inhibitor cocktail (Roche, 04693132001). After centrifugation at 20000*g* for 20 min at 4 °C, the supernatants were incubated with EGCG/RSVL-conjugated Sepharose 4B for 3 h at 4 °C. Proteins were analyzed by immunoblotting with indicated antibodies.

### Enzyme-linked immunosorbent assay

ARPE-19 cells were seeded into 12-well plates at a density of 2 × 10^5^ cells per well and treated as indicated. The secreted interferon in cell culture medium was analyzed with enzyme-linked immunosorbent assay (ELISA) kits (EHC026b.96, Neobioscience, for human) according to the manufacturer’s instruction.

### Quantification and statistical analysis

A standard two-tailed unpaired Student’s *t*-test was used for statistical analysis of two groups. Data are expressed as mean ± SEM. Graphs and statistical analysis were performed using GraphPad Prism (version 8.0). *P* values < 0.05 were considered as statistically significant. Flow cytometry data were analyzed by FlowJo (version 10).

## Data Availability

All data generated or analyzed during this study are included in this article.

## Abbreviations

RPE: Retinal pigmented epithelium
AMD: Age-related macular degeneration
cGAS: GMP-AMP (cGAMP) synthase
EGCG: Epigallocatechin gallate
RSVL: Resveratrol
GA: Geographic atrophy
VEGFA: Vascular endothelial growth factor A
IFNs: Type I interferons
mtDNA: Mitochondrial DNA
ELISA: Enzyme-linked immunosorbent assay
dsRNA: Double-stranded RNA
poly(I:C): Polyinosinic-polycytidylic acid
G3BP1: GTPase-activating protein SH3 domain–binding protein 1
SG: Stress granules
